# Natural clines and human management impact the genetic structure of Algerian honey bee populations

**DOI:** 10.1186/s12711-023-00864-5

**Published:** 2023-12-19

**Authors:** Giovanna Salvatore, Amira Chibani Bahi Amar, Kamila Canale-Tabet, Riad Fridi, Nacera Tabet Aoul, Soumia Saci, Emmanuelle Labarthe, Valentino Palombo, Mariasilvia D’Andrea, Alain Vignal, Pierre Faux

**Affiliations:** 1 Department of Agricultural, Environmental and Food Sciences, University of Molise, Via De Sanctis Snc, 86100 Campobasso, Italy; 2 Laboratoire de Génétique Moléculaire et Cellulaire (LGMC), Département de Génétique Moléculaire Appliquée, Université des Sciences et de la Technologie d’Oran Mohamed Boudiaf, USTOMB, BP 1505, El M’naouer, 31000 Oran, Algeria; 3 GenPhySE, Université de Toulouse, INRAE, INPT, INP-ENVT, 31326 Castanet-Tolosan, France; 4 Department of Biotechnology, Faculty SNV, University of Oran1 Ahmed Ben Bella, Oran, Algeria; 5 National Institute of Agronomic Research of Algeria (INRAA), El Harrach, Alger, Algeria

## Abstract

**Background:**

The Algerian honey bee population is composed of two described subspecies *A. m. intermissa* and *A. m. sahariensis*, of which little is known regarding population genomics, both in terms of genetic differentiation and of possible contamination by exogenous stock. Moreover, the phenotypic differences between the two subspecies are expected to translate into genetic differences and possible adaptation to heat and drought in *A. m. sahariensis*. To shed light on the structure of this population and to integrate these two subspecies in the growing dataset of available haploid drone sequences, we performed whole-genome sequencing of 151 haploid drones.

**Results:**

Integrated analysis of our drone sequences with a similar dataset of European reference populations did not detect any significant admixture in the Algerian honey bees. Interestingly, most of the genetic variation was not found between the *A. m. intermissa* and *A. m. sahariensis* subspecies; instead, two main genetic clusters were found along an East–West axis. We found that the correlation between genetic and geographic distances was higher in the Western cluster and that close-family relationships were mostly detected in the Eastern cluster, sometimes at long distances. In addition, we selected a panel of 96 ancestry-informative markers to decide whether a sampled bee is Algerian or not, and tested this panel in simulated cases of admixture.

**Conclusions:**

The differences between the two main genetic clusters suggest differential breeding management between eastern and western Algeria, with greater exchange of genetic material over long distances in the east. The lack of detected admixture events suggests that, unlike what is seen in many places worldwide, imports of queens from foreign countries do not seem to have occurred on a large scale in Algeria, a finding that is relevant for conservation purposes. In addition, the proposed panel of 96 markers was found effective to distinguish Algerian from European honey bees. Therefore, we conclude that applying this approach to other taxa is promising, in particular when genetic differentiation is difficult to capture.

**Supplementary Information:**

The online version contains supplementary material available at 10.1186/s12711-023-00864-5.

## Background

From a genetic standpoint, the honey bee *Apis mellifera* can be considered as a semi-domestic species. Indeed, although colonies are managed for the purpose of honey production or for pollination, the balance between natural reproduction and human control will vary according to the practices of the breeders, by extent influencing local population structures. In natural conditions, queens are usually inseminated by 15 to 30 drones from neighbouring colonies during mating flights [1], but breeders will often influence the genetic structure of populations through the trade of breeder queens, the use of mating stations that saturate the environment with drones of known origin, or even artificial insemination [2]. The purpose of genetic management in *Apis mellifera* can be to improve traits such as honey production, calmness or resistance to diseases. This can take the form of dedicated selection programs for general production traits [2] or for specific traits such as the production of Royal Jelly [3, 4]. The trade of honey bee queens is another form of genetic management, which is triggered either by a deficiency in local queen production failing to match colony losses or by the import of genetic stock having more desirable characteristics than the local populations. For instance, a population genomics study showed that *A. m. ligustica*, *A. m. carnica* and *A. m. caucasia* honey bee subspecies were imported to France, where they hybridized to the local *A. m. mellifera* or replaced it in some apiaries [5].

Over 30 *A. mellifera* subspecies are described worldwide [6], defined according to morphological, behavioural, physiological and ecological traits suited to their local habitat. Amongst these, two subspecies are found in Algeria: *A. m. intermissa*, the Tellian honey bee and *A. m. sahariensis*, found in the Sahara Desert [6]. Although there is now a substantial dataset of available phased haploid drone sequences that can be used for genetic diversity studies [7], little is known on these two subspecies and the genetic structure of their populations.

*A. m. intermissa* [8] is found in North Africa, extends across Morocco, Algeria and Tunisia, and is characterized by its small size, dark colour (see Additional file 1: Fig. S1), and aggressive defence behaviour [6]. It occupies the majority of Algerian apiaries, and is well adapted to important variations in climatic conditions [9]. In contrast, *A. m. sahariensis* is found in the southern part of Algeria and Morocco, ranging from the oases of the Sahara to the south of the Atlas Mountains [10]. This honey bee differs from the Tellian bee by the yellow body colour (see Additional file 1: Fig. S1) and a less defensive behaviour. It is able to adapt to extreme conditions such as high temperatures and drought conditions usually found in the Sahara [11]. In spite of this, *A. m. sahariensis* is facing several threats that are of anthropic and natural origins, such as the increased frequency of droughts in the Saharan steppes over the last decades, in addition to global warming which aggravates the already precarious situation of this subspecies [12].

Several studies have been carried out to characterize the two subspecies according to morphological and molecular aspects. The morphometric studies undertaken on Algerian honey bees have shown the existence of two distinct subspecies that morphologically correspond to *A. m. intermissa* and *A. m. sahariensis* [13–15]. Several molecular studies have been conducted on Algerian bees notably with microsatellite markers [16] and a single *A.m. intermissa* individual was sequenced [17]. In addition, research has also been performed on hive products (honey and pollen) in different scientific contexts [18].

Therefore, we produced haploid drone sequence data for 108 *A. m. intermissa* and 43 *A. m. sahariensis* samples to extend the haploid genomes dataset and to investigate several questions regarding Algerian honey bee populations. We first assessed the degree of admixture in Algerian honey bees resulting from the possible introduction of European honey bees from other subspecies, namely *A. m. mellifera*, *A. m. iberiensis*, *A. m. carnica*, *A. m. ligustica* and *A. m. caucasia*. Then, in order to better understand the structure of the Algerian honey bees, we focused on the Algerian samples, to investigate the genetic differences between the *A. m. intermissa* and *A. m. sahariensis* subspecies and the possible effects of honey bee management within the country. To this effect, we performed identical-by-descent (IBD) kinship analyses and correlations between genetic and geographic data. Finally, we performed a machine learning (ML) approach to select a reduced set of 96 single nucleotide polymorphisms (SNPs) with the aim of differentiating the Algerian subspecies from the European ones, to aid in conservation management. For such purposes, combining high-throughput sequencing and ML has the potential of making a significant impact on genetic characterization analysis across various taxa, as already shown for instance in cattle, pig, sheep and trout [19–22]. We also demonstrated the efficiency of our SNP set in a study simulating an F1 and several reciprocal backcross populations between Algerian and European genetic backgrounds. Testing the reduced panel of SNPs on an introgressed population can help to identify and differentiate between different levels of introgression, while also enabling more efficient and cost-effective assessment of genetic diversity and population structure for conservation purposes, catering to the needs of local beekeepers.

## Methods

### Sampling sites

In total, 151 drone samples, each one assumed to represent a single random colony, were collected from 20 regions in Algeria between 2017 and 2022. From 2017 to 2018, 91 adult drones, were collected at 14 sites [23]. Then from 2021 to 2022, 60 nymph drones were sampled at ten sites. There were four common sites between the two sampling campaigns. During the sampling, differentiation between the two native Algerian subspecies *A. m. intermissa* and *A. m. sahariensis* were based on the phenotype (see Additional file 1: Fig. S1): *A. m. intermissa* is described as a black honey bee naturally found in the north part of Algeria [8] and *A. m. sahariensis* as a yellow honey bee found in the south part of Algeria [10, 11]. *A. m. intermissa* samples (*N* = 108) were collected from 16 regions from the North West to the North East of Algeria (see Additional file 2: Table S1). *A. m. sahariensis* drones (*N* = 43) were sampled at five different sites located in the South of Algeria (see Additional file 2: Table S1). Spatial coordinates were reported for all sampling sites but not disclosed for privacy reasons. All the samples were preserved in absolute ethanol and stored at − 20 °C until DNA extraction.

### DNA extraction and sequencing

Total DNA was extracted from the head and thorax for adult samples, and from the whole body for individuals at the nymphal stage as described in Fridi et al., [23]. DNA sequencing was performed at the GeT-PlaGe core facility (INRAE Toulouse). DNA-seq libraries were prepared according to Illumina’s protocols using the Illumina TruSeq Nano DNA HT Library Prep Kit. Briefly, DNA was fragmented by sonication and adaptors were ligated for sequencing. The libraries were amplified for eight PCR cycles and quantified by qPCR using the Kapa Library Quantification Kit. Library quality was assessed using an Advanced Analytical Fragment Analyzer. DNA-seq experiments were performed on an Illumina NovaSeq 6000 S4 lane using a paired-end read length of 2 × 150 pb with the Illumina NovaSeq 6000 Reagent kits. Sequence reads were made publicly available after deposition on SRA (www.ncbi.nlm.nih.gov/sra/PRJNA1044268).

### Mapping and genotyping calling

Sequencing reads were mapped to the reference genome Amel_HAv3.1 [24] using the BWA-MEM (v0.7.15) software [25], as previously described by Wragg et al. [5]. Duplicates were marked with the PICARD MarkDuplicates tool (http://broadinstitute.github.io/picard/). Local realignment and base quality score recalibration (BQSR) were performed using GATK (version 4.1.2.) [26], with SNPs that were called with GATK HAPLOTYPECALLER as covariates for BQSR.

Each drone was processed independently with the pipeline and genotyped independently with HAPLOTYPECALLER. Although the sequenced drones are haploid, variant calling was performed using a diploid model to allow the detection and removal of SNPs for which heterozygous genotypes are called in more than 1% of samples, and that might have arisen for example as a result of short-tandem repeats (STR) or could highlight copy number variants (CNV) in the genome.

### Reference population

As a reference population we selected, from a previous study [5], the representative samples of the main evolutionary lineages, which are, (i) the M lineage (*A. m. mellifera* and *A. m. iberiensis*) (N = 63), (ii) the C lineage (*A. m. ligustica*, *A. m. carnica* and *Royal Jelly*) (*N* = 148) and (iii) the O lineage (*A. m. caucasia*) (*N* = 17). In practice, we selected the samples according to ADMIXTURE [27] results at K = 3, retaining the samples with a major ancestral proportion higher than 0.95. Individual genomic variant call format (gVCF) files from Algerian samples and individual gVCF from reference samples were combined with GATK COMBINEGVCFS and then jointly genotyped with GATK GENOTYPEGVCFS, resulting in a single VCF file for the 379 samples (228 reference samples and 151 Algerian samples) and containing 17,817,395 raw variants.

### SNP quality control

After discarding insertion-deletions (indels) with GATK SELECTVARIANTS, 12,302,217 SNPs were retained. Quality control (QC) on these SNPs was then performed as described in Wragg et al. [5]. Briefly, the first round of filters was applied to the entire dataset of 379 samples to address technical issues related to sequencing and alignment. These filters included (i) checks for strand biases and mapping quality metrics (stand odds ratio (SOR) ≥ 3; Fisher strand (FS) ≤ 60 and mapping quality (MQ) ≥ 40), (ii) genotyping quality metrics (SNP quality (QUAL) > 200 and quality depth (QD) < 20), and (iii) individual SNP genotyping metrics (heterozygote calls < 1%; missing genotypes < 5%, allele number < 4 and less than 20% of the samples with genotypes having an individual genotyping quality (GQ) < 10). The final dataset consisted of 8,865,912 SNPs.

### Identity-by-descent analyses

We used the program *hmmIBD* [28] to compute IBD kinships between pairs of samples. In brief, this program implements a hidden Markov model that aims at detecting IBD segments between pairs of haploid samples and was initially developed for the analysis of *P. falciparum*. Therefore parameters *nchrom* and *rec_rate* were respectively modified to 16 and 9.04 ×  10^–7^ to match *A. mellifera* specificities. We ran *hmmIBD* on a subset of 168 k randomly chosen SNPs (i.e. ~ 5% of the variants that were polymorphic within the Algerian population) to decrease the computational burden of this analysis. The computation of IBD kinships was first run on the initial 151 samples, in order to identify pairs of 1st degree related samples and remove these pairs (see section *Sample filtering*). However, as we found kinship estimates to be inflated in case of population structure, we ran again the kinship estimation on (1) the 102 retained samples (for assessment of genetic-geographic correlations—see dedicated section) and (2) the two main genetic clusters. The purpose of this latter kinship estimation was to compare the two clusters in terms of the number of closely-related pairs of samples; to that end, we focused on kinships up to the 3rd degree (~ 12.5%) hence above a conservative cut-off of 10%.

### Sample filtering

To avoid strong family substructure, we sought to identify and discard pairs of samples that were related at the first degree (IBD kinship ~ 50%—see section Identity-by-descent analyses). The distribution of IBD kinships showed a clear distinction between unrelated or far-related samples and samples likely to originate from the same queen (hence considered as full sibs; see Additional file 1: Fig. S2). Based on this distribution, we set the IBD threshold to 30% and identified 16 putative families of full sibs (from 2 to 26 samples per family; involving 64 samples—see Additional file 2: Table S1). Within each family, we retained only one sample (the sample with the best call rate), leaving 48 discarded samples. Call rates of the remaining 103 samples were all above 98.8% except for one sample (71.4%—see Additional file 2: Table S1) which was subsequently discarded, leaving a final number of 102 samples (86 *A. m. intermissa*, 16 *A. m. sahariensis*), from 48 sampling locations. We then pruned SNPs in strong linkage disequilibrium (LD) using the *indep-pairwise* function of the PLINK software [29] with a window size of 1749 SNPs, corresponding to a mean chromosome coverage of 100 kb (as detailed in Wragg et al. [5]). The LD pruning process involved a 10% overlap between windows, an LD threshold of 0.30 and a minor allelic frequency higher than 1%. The LD pruning was achieved independently after sample filtering for (1) the whole dataset (Algerian samples + reference samples; *N* = 330), leaving 1,305,303 SNPs, and (2) only the Algerian samples (*N* = 102), leaving 1,374,698 SNPs after LD pruning, which were the retained SNPs for subsequent analyses.

### PCA and admixture

Principal component analysis (PCA) was performed on the LD pruned dataset using SMARTPCA from the EIGENSOFT package version 7.2.1 [30]. In addition, Admixture analysis with reference samples and within Algerian samples was performed using the ADMIXTURE software version 1.3.0 [27], for *K* values ranging from 2 to 12. For each value of *K*, 50 Admixture runs were generated with different random seeds, according to Wragg et al. [5]. The most likely *K* was inferred from ADMIXTURE outputs using its default cross-validation (CV) method and based on the lowest CV error mean among the 50 runs. We used the program pong [31] to align the runs with different *K* values and group the results of the runs in the clustering mode.

### Correlations between genetic and geographical distances

We computed Pearson correlations between the top seven principal components (PC) and the three spatial coordinates. In addition, we obtained estimates of genetic distances between pairs of samples by computing the Euclidean distance on the top two PC, weighting the first one by the ratio between the first and second eigenvalues. Then, we computed the Pearson correlation between either these genetic distance estimates or the pairwise IBD kinships and the pairwise geographical distances, obtained using the Haversine formula (great-circle distance on a sphere). All geographic maps were produced using MATLAB ®.

### Random forest marker selection and validation procedures

A ML method, the random forest classifier (RF), was used to build a model that identifies ancestry informative markers (AIM) for assigning bee samples as Algerian or non-Algerian (M and C lineages—we kept the O lineage apart as too few reference samples were available). The RF classifier measures the importance of the features (SNPs in our case) and, using a criterion (either Gini impurity or entropy in our implementation) that measures the quality of tree building, assesses the role of each feature in the classification. This importance can be later used as an indicator of the informativeness of the SNPs. In our implementation, the ranking of the SNPs was performed based on two different feature selection methods, according to Schiavo et al. [20]: (1) SNPs that were more frequently present in the first list of the top 96 SNPs after 100 runs, and (2) SNPs with the highest significance in the average of 100 runs. As we tested both criteria (Gini impurity and entropy), the two methods led to four different configurations and thus four different SNP panels, “GI1” and “GI2” for Gini criterion and “EN1” and “EN2” for entropy criterion.

We fed the RF classifier with a total of 99,274 SNPs as AIM candidates. This reduced set of SNPs was obtained by retaining those with no missing genotypes and by strengthening LD filtering to reach a final number close to 100 k SNPs (plink *–indep-pairwise* value of 0.13). We trained the RF classifier to assign samples to their actual label (“Algerian” or “non-Algerian”) in two population-wise settings: (a) with 82 Algerian samples and 50 samples from the M lineage, and (b) with the same 82 Algerian samples and 118 samples from the C lineage. To average the results in terms of selected SNPs, the classification was achieved 100 times under each of the four configurations (GI1, GI2, EN1 and EN2) and for each population setting, using the *scikit-learn* Python environment [32] (function *RandomForestClasssifier*, with default parameters, except criterion set to “gini” or “entropy”). Then, considering that the most conventional microplate is a 96-well plate format, we retained as AIM the top 48 SNPs within each setting (Algerian versus C lineage and Algerian versus M lineage), leading to a panel of 96 SNPs for each configuration.

Each of the four panels was then trained to correctly assign the label of the 250 training samples and was further applied to 63 test samples (20 Algerian samples; 13 and 30 from the M- and C lineages respectively), i.e. samples that were involved neither in SNP selection, nor in population assignment. The panels were validated using PCA, to select the panel that maximizes the distance between Algerian samples and European lineages (C and M) while minimizing the distance between Algerian samples. The performances of the selected panel were eventually assessed by comparison with 1000 panels of 96 randomly-selected SNPs (among the reduced set of 99,274 SNPs). Each random panel was used 50 times to train a classifier and assign test samples. Then, we recorded, for each random panel, the average classification accuracy of the test samples as well as the minimum and maximum probability of being labelled “Algerian” respectively for Algerian and non-Algerian test samples.

### Simulated hybrids and RF validation

With the purpose of testing the performances of the candidate 96 AIM panel in a simulated admixture scenario, we created artificial hybrid and backcross genotypes using the *gscramble* R package (source code available at https://github.com/eriqande/gscramble). More in detail, 320 simulated genotypes were generated considering training (*N* = 250) and testing datasets (*N* = 63), separately. Forty random matings were chosen to generate offspring at each generation among all possible unique combinations between Algerian honey bees and individuals from the other lineages under investigation (i.e. M and C). The final simulated dataset included 640 hybrids: (i) 160 F1 progeny (80 for F1M and 80 for F1C), (ii) 160 first backcross (BC1) hybrids obtained from F1 x Algerian (80 for BC1M and 80 for BC1C), (iii) 160 second backcross (BC2) hybrids generated from BC1 x Algerian (80 for BC2M and 80 for BC2C), and (iv) 160 third backcross (BC3) hybrids generated from BC2 x Algerian (80 for BC3M and 80 for BC3C). Overall, the final dataset was merged with the original training and testing datasets and split in two new training (570 samples: 250 actual samples + 320 simulated hybrids) and testing datasets (383 individuals: 63 actual samples + 320 simulated hybrids). Two RF models were trained considering (1) the original training dataset (*N* = 250) and (2) the original training dataset plus hybrids (*N* = 570) by using the best performing panel of 96 AIMs. Models were both tested on the same dataset (*N* = 383). The resulting probability matrix of assignment to a class was compared with that obtained by using all variants.

## Results

### Population structure with reference subspecies

**Fig. 1:**
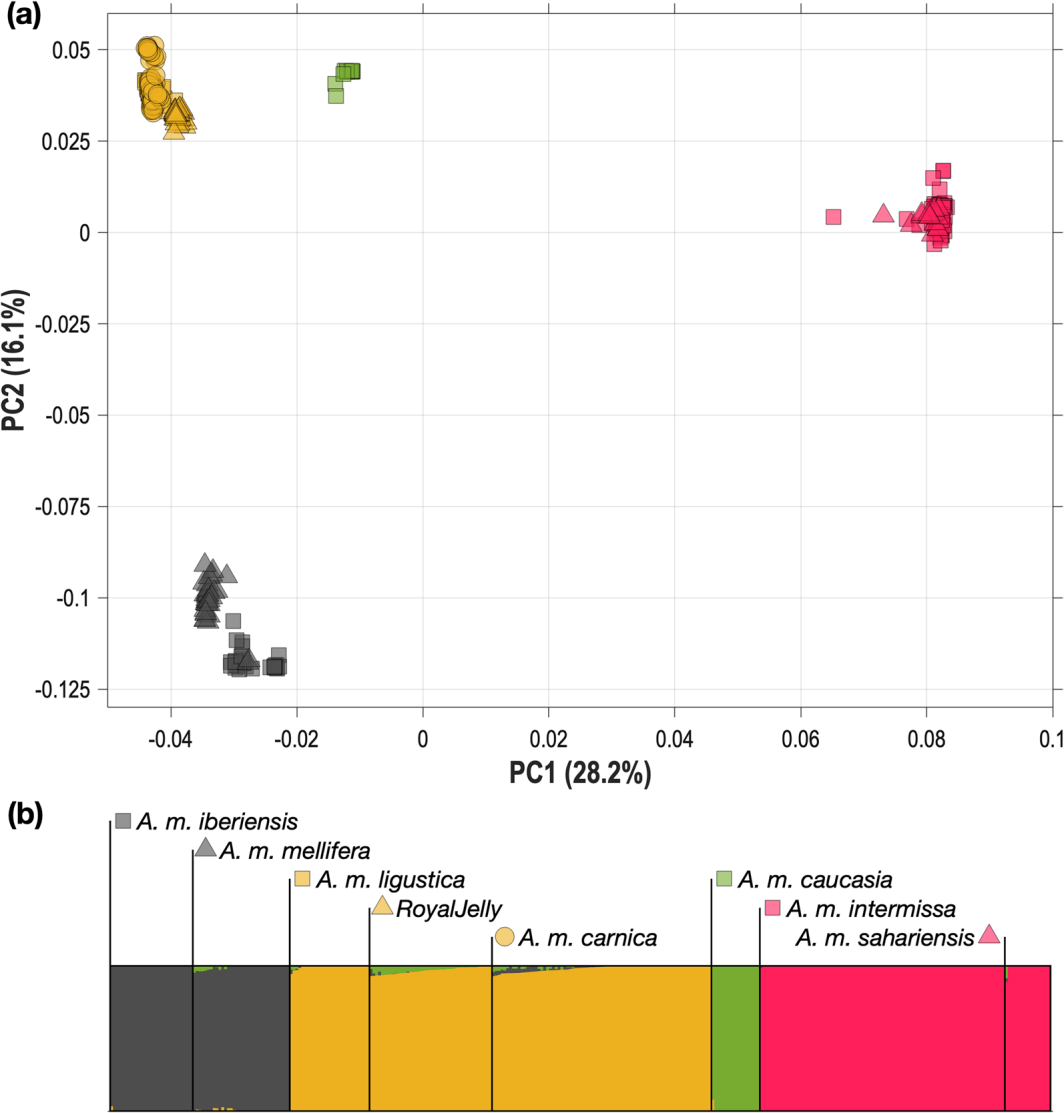
PCA and Admixture analyses over the European and Algerian populations. **a** PCA analysis plot of the top two principal components, showing the distribution of the two Algerian honey bee subspecies and the three main reference honey bee subspecies (samples are identified by marks in **b** legend). **b** Plot of ADMIXTURE (*K* = 4) results containing all the samples. Each box represents a different honey bee subspecies: *A. m. iberiensis* and *A. m. mellifera* (in grey), *A. m. ligustica*, *Royal Jelly*, *A. m. carnica* (in yellow), *A. m. caucasia* (in green) and Algerian samples (in red)

Initially, we examined the relationship of the reference populations (*N* = 228: *A. m. mellifera*, *A. m. iberiensis*, *A. m. carnica*, *A. m. ligustica* and *A. m. caucasia*) with the Algerian samples (*N* = 102: 86 *A. m. intermissa* and 16 *A. m. sahariensis*) using the 1,305,303 SNPs retained after all filtering steps. The first two genetic PC explain 28.23% and 16.07% of the variation, respectively. The PCA plot (Fig. 1a) showed a clear separation of honey bee populations into clusters that were largely consistent with their geographic origins. Populations from the M lineage (*A. m. mellifera* and *A. m. iberiensis*), C lineage (*A. m. ligustica, A. m. carnica* and *Royal Jelly*—a specifically selected honey bee, with a major component of *A. m. ligustica* [5]), O lineage (*A. m. caucasia*) and Algerian samples (*A. m. intermissa* and 16 *A. m. sahariensis*) formed distinct clusters. PC1 provides major separation of the European subspecies from the Algerian samples while PC2 distinguishes the M and C lineages in Europe.

Next, we assessed the degree of admixture in the reference and Algerian samples. To that end, we performed 50 runs of ADMIXTURE by varying *K* from 2 to 12 and found that *K* = 4 had the lowest CV error on average over all runs (see Additional file 1: Fig. S3). Figure 1b shows that these four clusters clearly correspond to the four lineages: (1) M lineage (*A. m. mellifera and A. m. iberiensis*), (2) C lineage (*A. m. ligustica, A. m. carnica* and *Royal Jelly*), (3) O lineage (*A. m. caucasia*), and (4) Algerian samples (*A. m. intermissa* and *A. m. sahariensis*).

### Population structure within Algerian subspecies

**Fig. 2:**
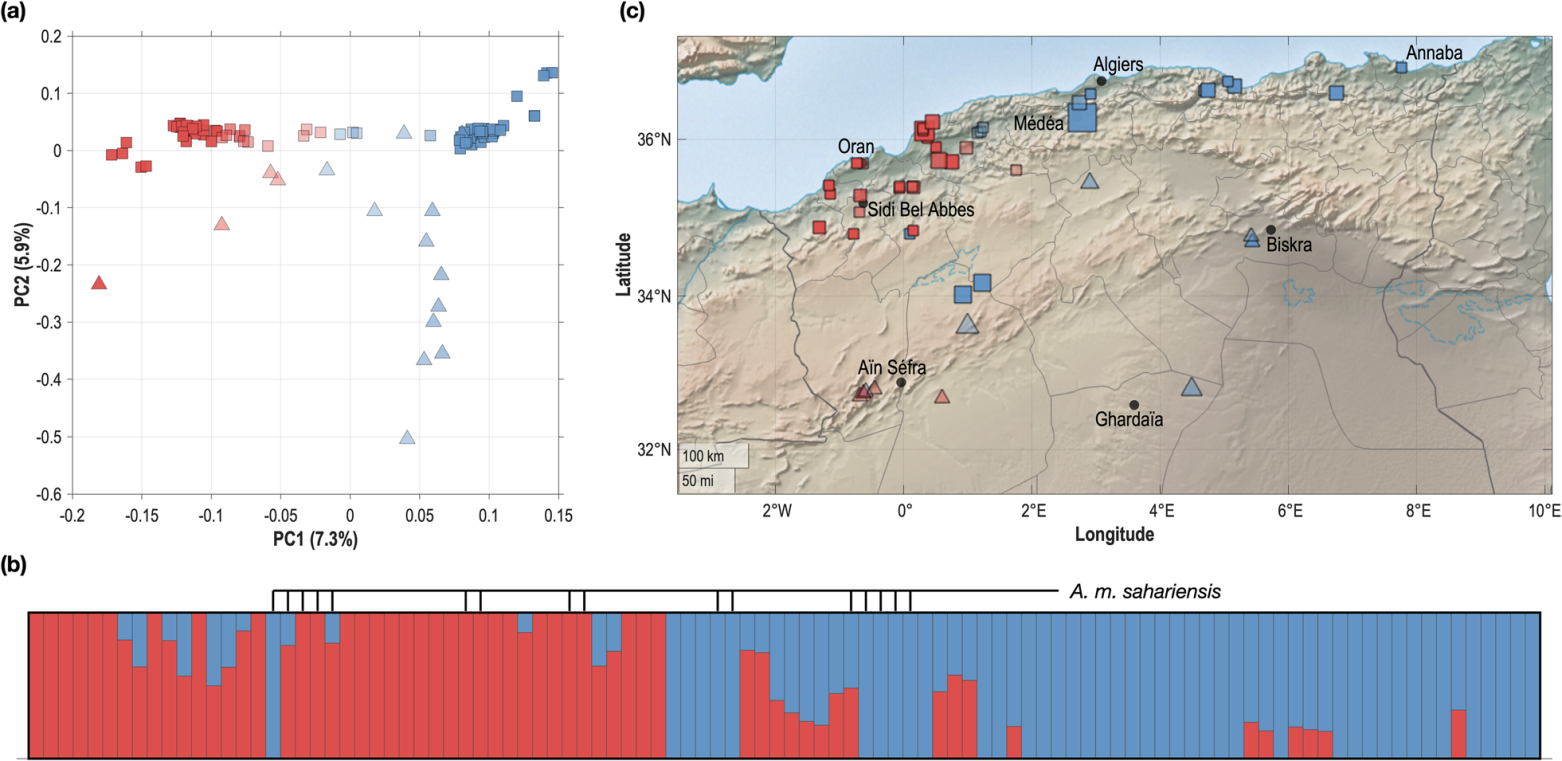
PCA and Admixture analyses restricted to the Algerian population and geographic distribution of samples. **a** PCA plot of the top two principal components (PC1 and PC2), over the Algerian honey bee population. Individual samples are coloured in accordance with their main ADMIXTURE component at *K* = 2, mark transparency indicating the most admixed samples and mark shapes denoting the subspecies (squares for *A. m. intermissa*; triangles for *A. m. sahariensis*). **b** Plot of ADMIXTURE results at *K* = 2 over the Algerian honey bee population, ordered by longitude. Each bar represents an individual sample and the 16 *A. m. sahariensis* samples are identified. **c** Distribution of sampling sites, using the same graphical code as in **a**, with admixture proportions averaged over all individuals sampled at a given site. The mark size is proportional to the number of samples

The same aforementioned analyses were restricted to the Algerian population (102 samples; 1,374,698 SNPs retained after filtering) in order to highlight the genetic structure within Algerian subspecies. The results are shown in Fig. 2a: the first two genetic PC, respectively, explain 7.29 and 5.94% of the total variation in these data. The first PC reveals a clear geographical cline, with specimens from western areas clustering toward the left of the plot and specimens from the eastern areas clustering towards the right. The two subspecies, *A. m. sahariensis* and *A. m. intermissa* (respectively, triangles and square in Fig. 2a), are discriminated along the second than rather the first axis.

The ADMIXTURE results indicate that the optimal number of populations, as determined by the CV error, is *K* = 2 (see Additional file 1: Fig. S4). We assigned the samples to their main ADMIXTURE component and coloured them in accordance with Fig. 2a, to stress how the ADMIXTURE clustering is captured by PC1. In Fig. 2b, we ordered them according to their longitude coordinates, hence along a West–East cline, a display that highlights the genetic differentiation between western and eastern samples observed in the PCA plot. Notably, a similar admixture plot was obtained when running ADMIXTURE with *K* = 5 on the European and Algerian bees (see Additional file 1: Fig. S3). The correlation between average proportions of corresponding admixture components (estimated either only on Algerian samples—for *K* = 2, or on European and Algerian samples—for *K* = 5), computed over the 102 Algerian samples, was equal to 99.8%, providing additional evidence on the existence of these two Algerian genetic backgrounds.

### Genetic-geographic correlations

In Fig. 2c, we show the geographical distribution of the samples, which suggests that it mirrors the PCA plot in Fig. 2a. Given this strong geographical pattern within the genetic structure of Algerian bees, we sought to correlate genetic and geographic variation. In Table 1, we assessed the Pearson correlation between the top genetic PC and the three spatial coordinates. This assessment was limited to the top seven PC as there was no nominally-significant correlation beyond the 6th PC. As expected from Fig. 2, the strongest correlation (*r* = 72%) was found between PC1 and longitude. The correlation with latitude was highly significant with the two subsequent PC (PC2 and PC3). We also noticed a highly significant correlation between PC3 and altitude that likely results from the correlation between altitude and latitude (r = − 61.6%) over the sampling area (the southernmost, the furthest from the sea hence the highest).

**Table 1:** Correlations between the top seven genetic principal components and the three spatial coordinates (*N* = 102 individual samples)

PC	Latitude (%)		Longitude (%)		Altitude (%)	
1	− 0.5		72.0	^(***)^	34.4	^(***)^
2	− 42.8	^(***)^	− 5.9		19.3	
3	− 54.2	^(***)^	− 35.7	^(***)^	43.3	^(***)^
4	4.4		− 1.6		− 9.7	
5	28.5	^(**)^	− 3.6		8.5	
6	− 23.6	^(*)^	26.1	^(**)^	− 10.2	
7	2.5		15.8		− 9.5	

Significance indicated by asterisks: ***P < 0.1%, **P < 1%, *P < 5%

We then assessed how genetic (dis-)similarity between a pair of samples relates to geographic distance between their sampling sites (Table 2). Using all pairwise geographic distances, we obtained highly significant correlations with either the genetic distance (measured as the Euclidean distance between pairs on the two first PC space – *r* = 45%) or the IBD kinship (*r* = − 26%). Interestingly, the positive correlation between genetic and geographic distances was mostly driven by the Western cluster, for which the correlation is almost three times that of the Eastern cluster. Scatter plots of these distances (see Additional file 1: Fig. S5) clearly show that, in the Eastern cluster, genetically-close individuals that are sampled at distant locations and genetically-distant individuals that are sampled at proximate locations decrease the correlation.

**Table 2:** Correlations between genetic distance or kinship and geographic distance

	*N*	*r* (%)	*r*^*2*^	*P*
*Using PC-based genetic distance*				
All samples	5151	45.0	0.202	9 × 10^–255^
Western cluster	990	71.6	0.512	4 × 10^–156^
Eastern cluster	1596	25.9	0.067	6 × 10^–26^
Between clusters	2565	30.7	0.094	4 × 10^–57^
*Using IBD kinship*				
All samples	5151	− 25.5	0.065	4 × 10^–77^
Western cluster	990	− 63.1	0.398	4 × 10^–111^
Eastern cluster	1596	− 21.3	0.045	8 × 10^–18^
Between clusters	2565	− 22.0	0.048	2 × 10^–29^

### Comparison of IBD kinship between main clusters

**Fig. 3:**
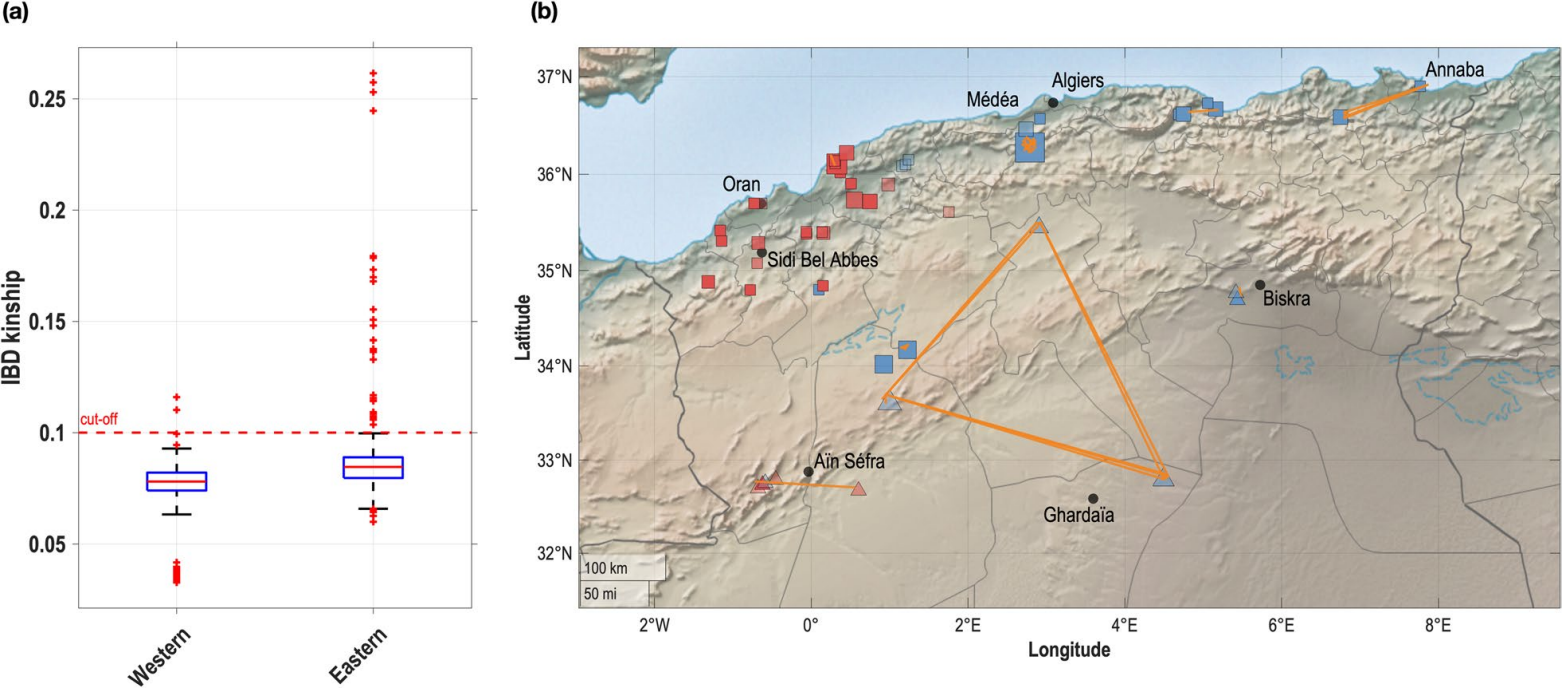
IBD kinship distribution in the sampling of the Algerian honey bee population. **a** Boxplot displaying the distribution of IBD kinship within each main cluster: the red horizontal bars show the median, the blue box covers 75% of the data, the whiskers extend to average plus three standard deviations and the red marks indicate outliers. The dashed horizontal red line indicates an IBD kinship of 10%, hence pairs of samples above this cut-off are related at least at the 3rd degree. **b** Geographic location of close-family ties (orange lines: pairs of samples with IBD kinship > 10%). Sampling sites are denoted by marks following the same drawing codes as in Fig. 2c

Using a program dedicated to IBD inference in haploid samples, we estimated IBD kinships between all pairs of samples. Considering as closely-related any pair of samples related up to the 3rd degree, we conservatively retained any pair for which the kinship was higher than 10%. Thirty-three pairs (over 5151 possible pairs) were identified as such (Fig. 3a): the vast majority of these pairs (31/33) involved samples from the Eastern genetic cluster, with values clustered around the 2nd (IBD kinship = 25%) and 3rd degrees (IBD kinship = 12.5%). In contrast, such a level of close-family relationships was not observed in the Western cluster (only two values above the cut-off: 11.0 and 11.6%, within the range of expected values of a 3rd degree relationship). Then, we identified the sampling locations involved in these close-family ties, which are depicted by orange lines in Fig. 3b. This geographical display shows that some close-family ties are found at large distances, mostly between the *A. m. sahariensis* samples.

### Selection of a panel of 96 ancestry-informative markers

**Fig. 4:**
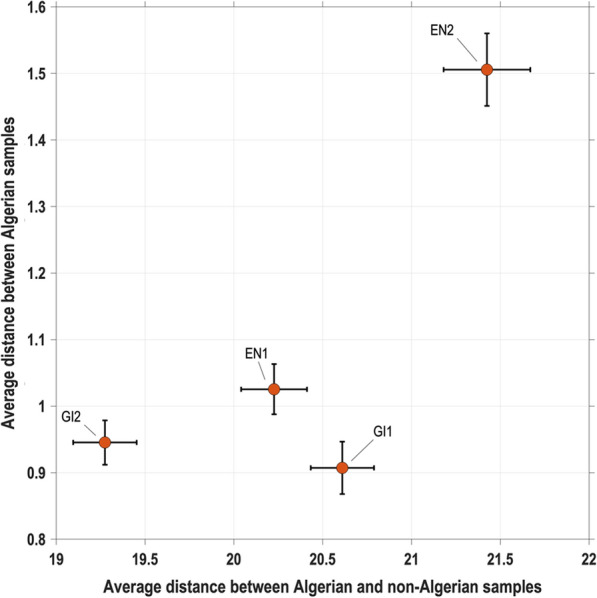
Comparison of the four panels of 96 selected SNPs by using pairwise distances between samples. Distances are computed using the two first principal components. Red dots indicate the average over 63 test samples, with standard errors shown by the whiskers

RF classifiers were trained to discriminate Algerian samples from non-Algerian samples. We repeated the classification a hundred times for each of the four configurations of feature selection (GI1, GI2, EN1 and EN2) and for each of the two population settings (Algerian bees vs. M or C lineage). This way, we established four panels of 96 SNPs, each of them including 48 SNPs that discriminate Algerian bees from the M lineage and 48 SNPs discriminating from the C lineage. In total, the four panels include 200 different SNPs. These SNPs are ranked over all the panels in Additional file 2: Table S2. In order to compare these panels, a validation step was performed on the test dataset (20% of original dataset, excluded from the AIM selection analysis) using PCA. The results are shown in Additional file 1: Fig. S6; the four panels distinguish the three lineages on the two first PC, which explain a much larger variance than the subsequent components. Thus, we used pairwise distances computed on these two first PC to identify the best panel. The purpose was to minimize the intra-Algerian pairwise distance and maximize the pairwise distance between Algerian and C-M lineage samples. In Fig. 4, plotting the averages of these two objectives against each other for the four SNP panels, we considered that the best configuration of all methods was GI1, hence a configuration in which the decision tree building uses Gini impurity and the frequency of a SNP among the top features over repeated runs is preferred to its highest significance (see Methods).

We further sought to assess how good our selection of 96 SNPs was, by comparing it to a random selection of the candidate features. To that end, we randomly selected 1000 panels of 96 SNPs (among the reduced set of 99,274 SNPs), trained a RF classifier 50 times using the same training population as for the selected panel, applied these classifiers to the test population, and recorded three parameters (average classification accuracy of test samples, minimum and maximum probabilities of being labelled “Algerian”, respectively for the Algerian and non-Algerian bees). This allowed us to draw the empirical distribution of these three parameters (see Additional file 1: Fig. S7). Over 50 repeated runs of classification of the test samples, the selected panel (GI1) always achieved a perfect classification (test accuracy = 100%) and got minimal/maximal probabilities of being labelled “Algerian” equal to 0.79 and 0.18, respectively, for Algerian/non-Algerian samples, which outperformed the 1000 random SNP panels.

### Testing the selected panel of AIM on simulated hybrids

To further assess the applicability of the panel of 96 AIM to non-observed samples, we generated artificial hybrids (by mating Algerian bees and samples from either the M or C lineages) and backcrosses from the 2nd to the 4th generation (by mating the hybrids and subsequent backcrosses with Algerian bees). We first trained a RF classifier using the original 250 samples and tested it on 320 simulated hybrids. In this setting, we found that all hybrids were classified as ‘Algerian’, with an increasing probability of being labelled as “Algerian” throughout generations (see Additional file 1: Fig. S8). Then, we trained a RF classifier with a dataset that included simulated hybrids of various generations. Note that those simulated hybrids were generated from samples of the training set itself and labelled as non-Algerians. Hence, here we mimicked a training population that included identified cases of recent admixture. In such a setting, which was repeated over 50 runs, we found that the classification accuracy remained quite high (99%) and that misclassifications occurred only on the 2nd or 3rd backcrosses from the M lineage (BC2M: 1.4% and BC3M: 7.25%—see Additional file 1: Fig. S9), or Algerian bees (0,8%).

**Fig. 5:**
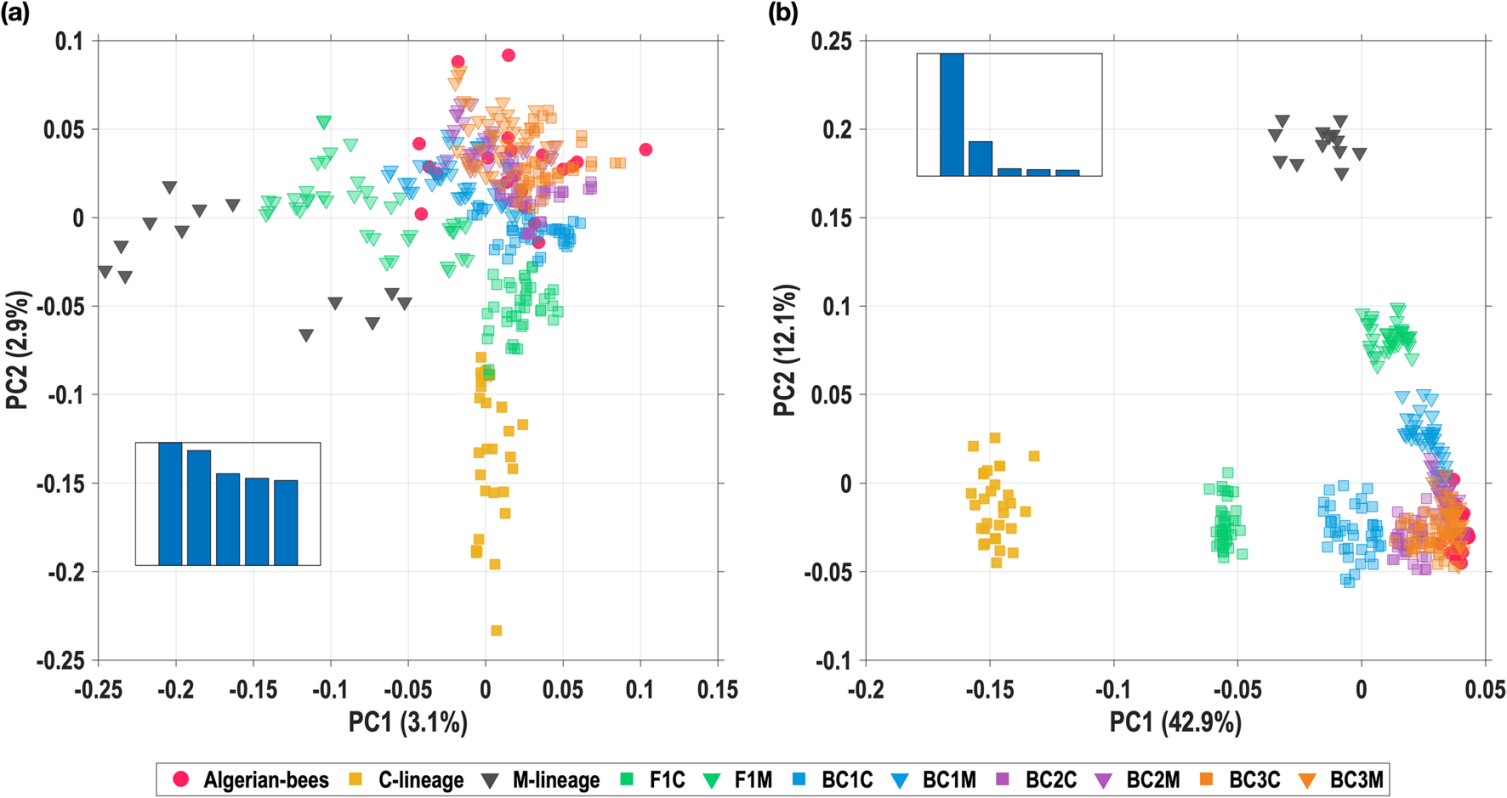
Principal component analysis of the test population including simulated hybrids, using either **a** 99,274 SNPs or **b** 96 selected AIM. The bar plot insets show the relative proportion of variance explained by the top-5 PC in each case

Finally, we checked whether these misclassifications arose from the reduced representativity induced by the use of 96 SNPs, or from the close relationship between the hybrids and the Algerian bees. To that end, we repeated 50 times the second setting described above (i.e. a training set including simulated hybrids) using all the 99,274 SNPs retained for AIM selection. We found that the 96 selected SNPs resulted in a higher mean probability of classification of Algerian samples as “Algerian” and a lower mean probability of classification of all the non-Algerian and hybrids as “non-Algerian” (except for BC2M and BC3M—see Additional file 1: Fig. S9), which suggests that these 96 SNPs form an effective selection of SNPs compared to the whole genome. This observation was further confirmed by comparing the PCA using either all the 99 k SNPs (Fig. 5a) or the 96 selected SNPs (Fig. 5b), which showed that the clusters of original and hybrid populations are distinguished in a finer way on the first two PC with the AIM panel.

## Discussion

The findings of this study provide a new contribution to our understanding of honey bee subspecies in Central-North Algeria. The samples were compared to reference populations that are representative of the three main evolutionary lineages, the M lineage (*A. m. mellifera* and *A. m. iberiensis*), the C lineage (*A. m. ligustica*, *A. m. carnica* and *Royal Jelly*) and the O lineage (*A. m. caucasia*)*.* While the two Algerian subspecies are geographically isolated from European honey bees, they have been in contact due to human-mediated introductions of European honey bees, as reported by local beekeepers and assessed by Loucif-Ayad et al. in 2015 [33]. In spite of this contact, the genetic analysis conducted here did not reveal evidence of substantial genetic introgression between the Algerian subspecies and European honey bees, as shown in Fig. 1: in both the PC and Admixture analyses, and Algerian bees were found to cluster tightly together and to stand clearly apart from the reference samples.

To some extent, this absence of recent introgression is also supported by our application of the panel of 96 AIM to simulated hybrids: when hybrids generated by recent admixture (up to the 4th generation) were included in the training set, test hybrids were almost always labelled as non-Algerians. Thus, if some of the Algerian samples were recently admixed, we would have expected them to behave in a similar way to these simulated hybrids, that is, being labelled as non-Algerians when the training set does not include hybrids. However, none of our analyses eliminates the possibility of a more ancient European admixture; thus, further investigations are needed to assess this point.

It has been established by previous studies that the two Algerian subspecies (*A. m. intermissa* and *A. m. sahariensis*) can be differentiated based on morphological and behavioural differences [6] and by mitochondrial, microsatellites and sequencing analyses [13–17]. Our sampling for this study includes both subspecies, although in a rather unbalanced way (16 *sahariensis* vs. 86 *intermissa*). Yet, our whole-genome genetic analysis did not reveal a clear differentiation between them. The second PC of the Algerian sampling is somewhat discriminant between subspecies (Fig. 2a) but confounds with latitude (significant negative correlation—Table 1), hence this PC shows a genetic gradient along a North–South cline rather than a clear separation between subspecies.

Instead of an interspecific clustering, we found that the main axis of genetic differentiation was a geographic cline, from the eastern to the western part of the study area (Fig. 2c). Interestingly, we found that these two main clusters behaved differently in both dispersal pattern and IBD analyses. In the Western cluster, genetic and geographic distances are well correlated, indicating that genetic proximity is limited to the immediate vicinity. In contrast, this correlation, although still positive, is much lower in the Eastern cluster: genetically-similar samples are found at long distances and geographically-close samples are not always related. In addition, the vast majority (> 93%) of the close family relationships (IBD kinships up to the 3rd degree) are found in that cluster, sometimes at long distances, as suggested in Fig. 3b. These differential results on both clusters are consistent with patterns of beekeeping practices such as commercial breeding or, with a lower impact, swarming during transhumance. Therefore, we hypothesize that the longitudinal cline is the main differentiation factor in the Algerian population and may be reinforced by differential breeding practices between western and eastern Algeria. In western Algeria, the population evolves either in a natural way (i.e. free-range mating) or through queen trades between beekeepers at a local level. In contrast, our results suggest that a third type of genetic exchange, at a long distance, takes place in eastern Algerian along with the two afore-mentioned ones. In that respect, the Algerian honey bee population offers a nice illustration of a species ranging from natural to domesticated behaviour. However, our study does not provide information on the factors that have contributed to the geographical differentiation. Additional research, including more representative samples of *A. m. sahariensis,* is needed to investigate the historical and ecological factors that have shaped the genetic structure of honey bee populations in Algeria.

The use of an RF approach has proven effective to achieve the selection of a 96 AIM panel that can distinguish between Algerian and non-Algerian samples, in our case C and M lineages (i.e. the most widespread and exported lineages). This panel is relevant for conservation purposes. For example, the development of a customized multiplex PCR panel to run massive genotyping based on simple amplicon sequencing for the SNaPshot^TM^ or MASSarray® platform could be explored, highlighting the potential economic advantages of using reduced SNP panels instead of dense genome-wide assays. This approach presents a viable option for future studies due to its potential application in distinguishing honey bee subspecies. Thus, we aimed at confronting its use in simulated cases of recent admixture. Our results on these simulated hybrids provided insights on the applicability of the 96 AIM panel and we found that it was effective to detect recent cases of admixture as long as (1) admixed samples identified as such are present in the training set, and (2) admixture is relatively recent. In our tests, the classification was perfect up to the 2nd generation when crossing with the M lineage and up to the 4th generation with the C lineage, a type of admixture that is more likely to occur [34]. Also of interest, the comparison of the selected GI1 list with all the variants (see Additional file 1: Fig. S9) showed a better classification performance when using 96 SNPs. ML algorithms may struggle to analyse data with a large number of features, a phenomenon known as the ‘curse of dimensionality.’ As the number of features increases, the ability of the algorithm to accurately predict outcomes may decrease [35].

These results have important implications for the management and conservation of honey bee subspecies in Algeria. The use of a ML approach, such as RF, to identify informative markers has broader implications for biodiversity research. This approach can be used to identify informative markers in other taxa and may be particularly useful for species or populations in which genetic differentiation is subtle or difficult to detect using traditional methods.

## Conclusions

Our research focused on unravelling the structure of the Algerian honey bee population, with particular attention paid to two distinct subspecies: *A. m. intermissa* and *A. m. sahariensis*. However, we did not find discernible genetic differentiation based on subspecies throughout our analyses. Instead, we found that geography played a significant role in shaping the genetic makeup of these bees, with distinct differences in population behaviour between the western and eastern areas. These variations may be driven by differences in breeding management; further research is however required to support that interpretation. In addition, we did not detect any recent introgression of European bees into the Algerian population, a finding of relevance for beekeeping activities and conservation policies. For this particular purpose, we also proposed a panel of AIM to classify Algerian vs. European bees. This approach can be helpful to ensure the long-term survival of native honey bee subspecies in Algeria. Overall, our study contributes to a better understanding of the genetic diversity and conservation needs of honey bees in Algeria and underlines the importance of preserving the unique subspecies found in this country.

### Supplementary Information

**Additional file 1: Figure S1.** Pictures of *A. m. sahariensis* (a) and *A. m. intermissa* (b). **Figure S2.** Distribution of IBD kinship between pairs of Algerian samples. The distribution of the values of IBD kinship over 11,325 pairs of Algerian samples (N = 151) is shown in this figure. The Y axis gives the number of pairs in each bin on a logarithmic scale (a value of 1, 2 or 3 respectively means 1, 10 or 100 pairs in the corresponding bin). The red dashed line indicates the retained cut-off (0.3) to define close-related samples. For pairs beyond that cut-off value, we retained only one member in the final set (see Additional file 2: Table S1). **Figure S3.** Estimation of cross-validation (CV) error for 50 runs of ADMIXTURE for 2 ≤ *K* ≤ 12, considering Algerian and reference specimens. Among the *K* values with the lowest CV values, *K* = 4 stands out as having a significant mode that comprises 48 out of 50 runs. This mode also has the lowest mean CV value from the ADMIXTURE runs. The admixture plot for K = 4 is given along with plots for 2 ≤ *K* ≤ 6. **Figure S4.** Estimation of cross-validation (CV) error for 50 runs of ADMIXTURE for 2 ≤ *K* ≤ 12, only considering Algerian specimens. **Figure S5.** Scatter plots of genetic vs. geographic pairwise distances. The top plot displays genetic (measured as the Euclidean distance on the two first PC) and geographic distances over all pairs of samples (N = 5151). In the bottom plots, all pairs are partitioned on their type: (a) both samples from the Western cluster, (b) each sample from a different cluster, and (c) both samples from the Eastern cluster. **Figure S6.** PCA of the test population using each of the four panels of 96 selected SNPs. The four PCA plots show the distribution of the 63 test samples (20 Algerian bees + 13 M lineage + 30 C lineage) on the first two principal components obtained using the 96 selected SNPs with each configuration (from top to bottom, left to right: GI1, GI2, EN1 and EN2). The four scree plots correspond to the each of four PCA. In all cases the top-2 PC explain a much larger proportion of variance than the subsequent PC. **Figure S7.** Prediction performance of the list of 96 selected SNPs against 1000 lists of randomly-chosen SNPs. We compared the selected panel of 96 SNPs (GI1) to 1000 randomly-selected SNPs. After using each of these random panels to repeat 50 times the training of a RF classifier ({Algerian; non-Algerian}) and its application to assignment of the 63 test samples, we recorded three parameters: (a) the average classification accuracy of the test samples over the 50 runs, (b) the minimum probability of being labelled Algerian for Algerian bees, and (c) the maximum probability of being labelled Algerian for non-Algerian bees. The values of these parameters obtained with the selected panel of 96 SNPs are shown in red below each histogram. **Figure S8.** Assignment probability of Algerian bees and simulated hybrids. We report the probability of a test sample being labelled “Algerian” by a random forest classifier for which the training set did not include simulated hybrids, for 20 Algerian bees and 320 simulated hybrids: four types of crosses (F1 and three back-crosses – BC) on 40 per type of cross. **Figure S9.** Assignment probabilities, using the panel of 96 AIM vs. using all variants. We report the probability of a test sample being labelled “Algerian” by a random forest classifier trained on a population that includes simulated hybrids, using either all variants (blue boxes) or the 96 selected AIM (yellow boxes), per population or per type of hybrid.

**Additional file 2: Table S1.** List of samples, with description, selection filters and SRA accessions. We identified 16 families of putative fullsibs: samples pertaining to these families are identified by the same family ID. Within each family, only the sample with the highest call rate was retained. In addition, one sample (shown in red) with a low call rate was not retained, leaving a final number of 102 retained samples. DNA sequences have been deposited in the Sequence Read Archive (SRA; at www.ncbi.nlm.nih.gov/sra) under the BioProject accession PRJNA1044268 (individual and run accessions in dedicated columns of this table). **Table S2.** Importance rankings of the 96 SNPs selected by four random forest criteria. In total, 200 different SNPs were selected by at least one method; 20 of them appearing in all four lists. If a given SNP was not selected by a given method, it was set to rank 97. Cells are coloured conditioning on rank (top ranks in red; bottom ranks in blue) and rows are ordered on total rank.

## Data Availability

DNA sequences for this study have been deposited in the Sequence Read Archive (SRA; at www.ncbi.nlm.nih.gov/sra/PRJNA1044268) under the BioProject accession PRJNA1044268. Please refer to Additional file 2: Table S1 for individual accessions.
