# Supplementary material for: Natural clines and human management impact the genetic structure of Algerian honey bee populations
Source: Genet Sel Evol. 2023 Dec 19;55:94. doi: 10.1186/s12711-023-00864-5 (PMC10729559; doi:10.1186/s12711-023-00864-5)

***“Natural clines and human management impact the genetic structure of Algerian honey bee populations”***

**Additional Figures**

## Figure S1. Pictures of a) *A. m. sahariensis* and b) *A. m. intermissa*


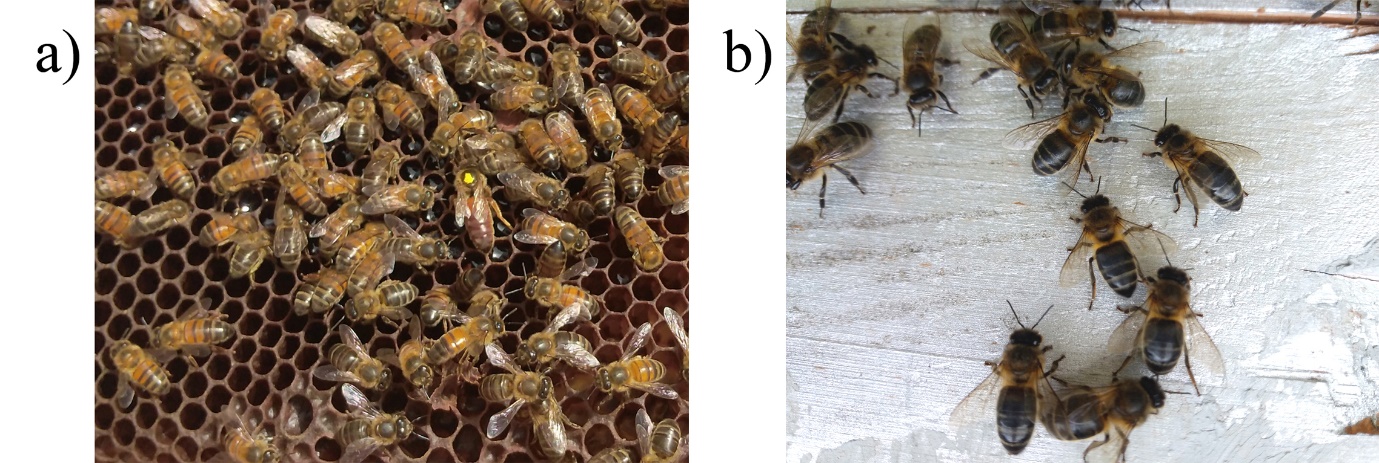


## Figure S2. Distribution of IBD kinship between pairs of Algerian samples

The distribution of the values of IBD kinship over 11,325 pairs of Algerian samples (N = 151) is shown in the figure below. The Y axis gives the number of pairs in each bin on a logarithmic scale (a value of 1, 2 or 3 respectively means 1, 10 or 100 pairs in the corresponding bin). The red dashed line indicates the retained cut-off (0.3) to define close-related samples. For pairs beyond that cut-off value, we retained only one member in the final set (see Supplementary Table Y1).


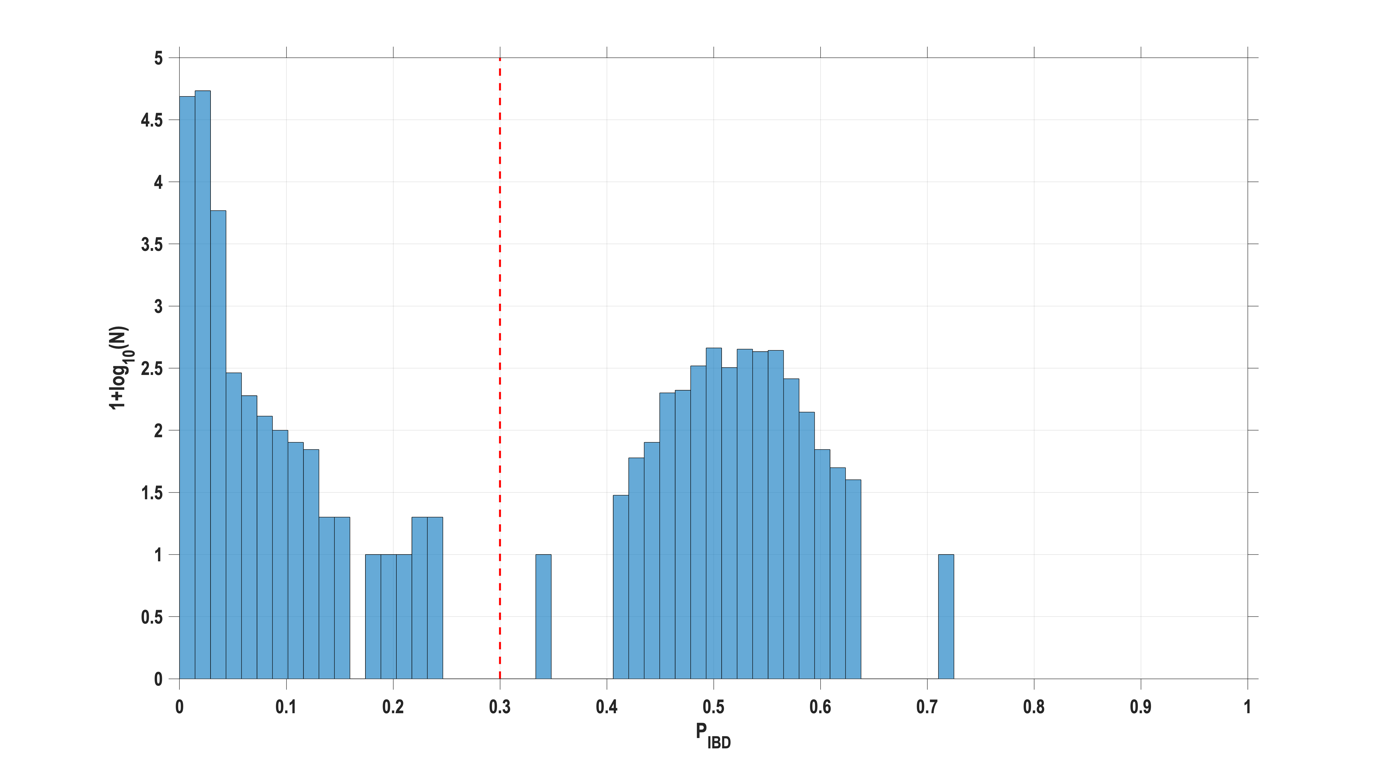


## Figure S3. Estimation of cross-validation (CV) error for 50 runs of ADMIXTURE for 2 ≤ K ≤ 12, considering Algerian and reference specimens

Among the K values with the lowest CV values, K=4 stands out as having a significant mode that comprises 48 out of 50 runs. This mode also has the lowest mean CV value from the ADMIXTURE runs. The admixture plot for K = 4 is given below, along with plots for 2≤K≤6.


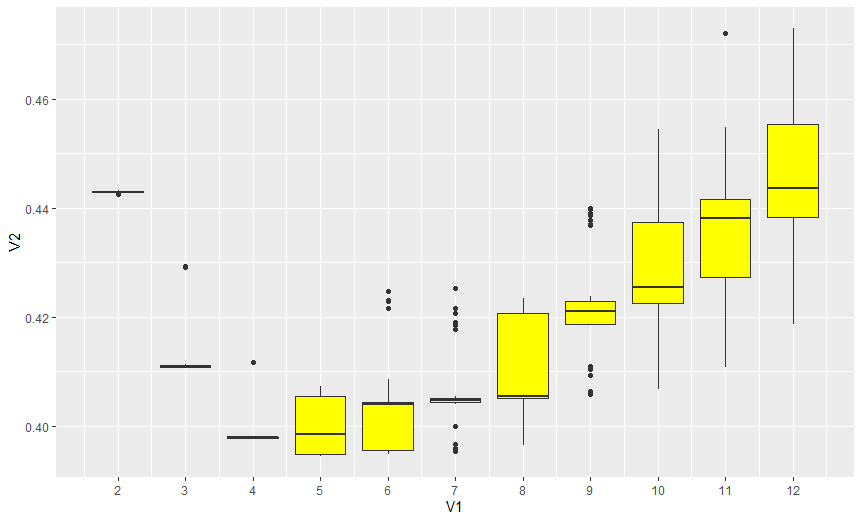


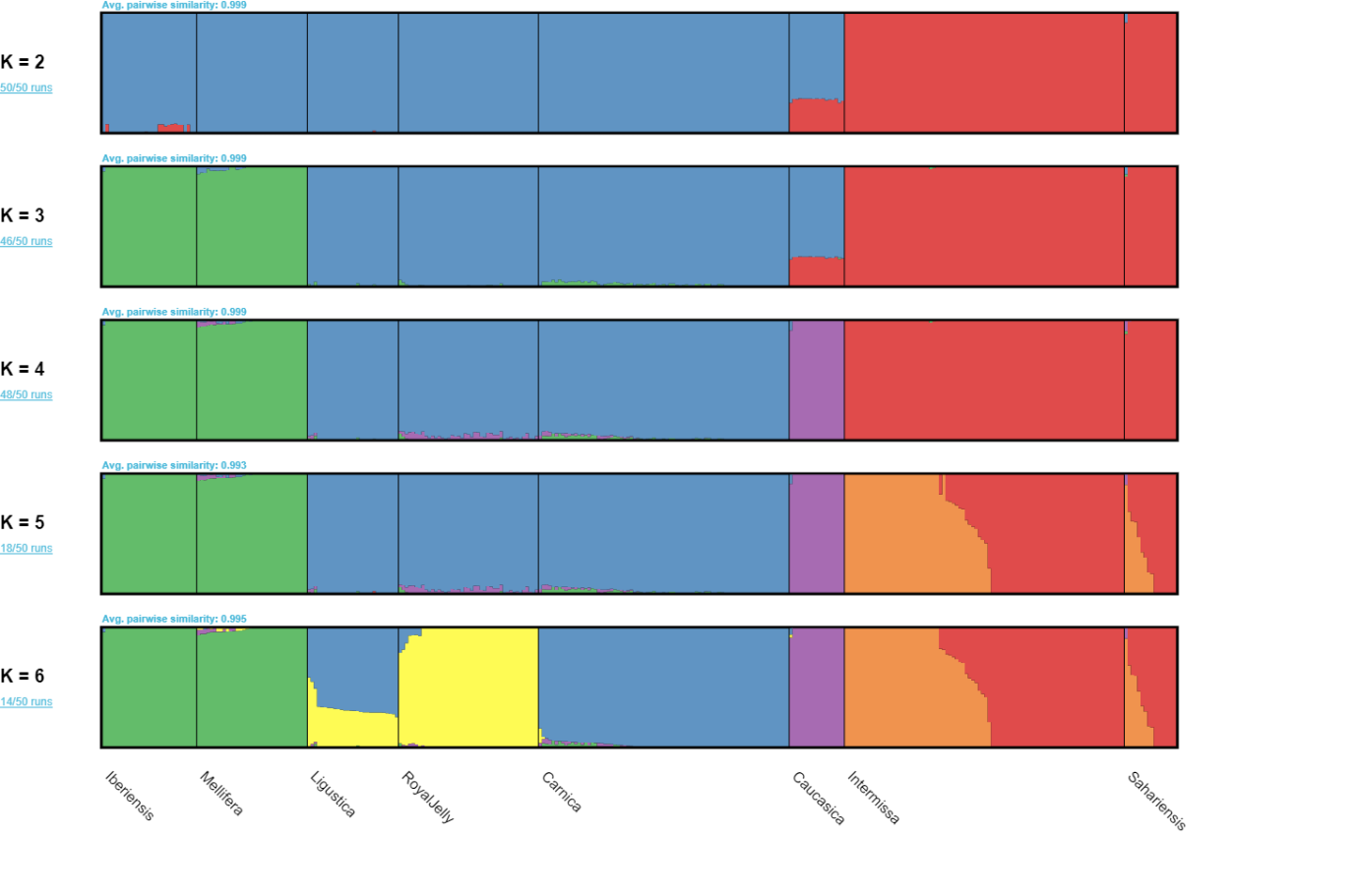


## Figure S4. Estimation of cross-validation (CV) error for 50 runs of ADMIXTURE for 2 ≤ K ≤ 12, only considering Algerian specimens

K=2 was chosen for subsequent analysis.


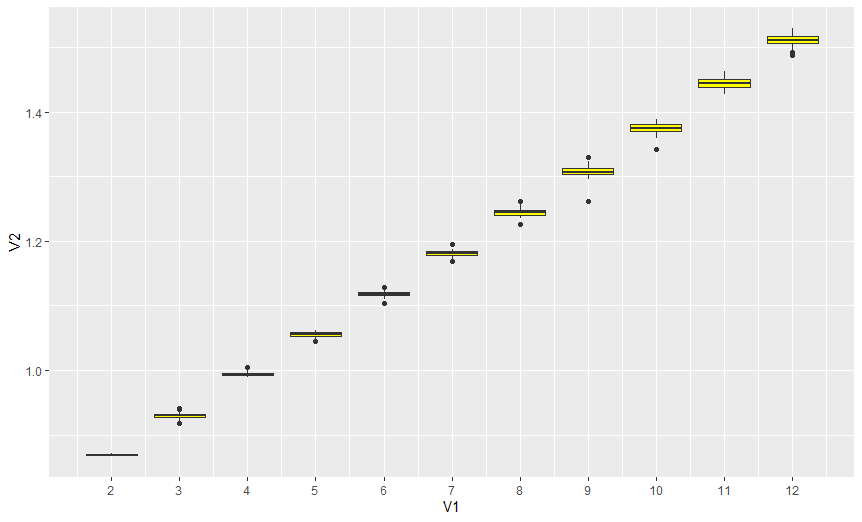


## Figure S5. Scatter plots of genetic vs. geographic pairwise distances

The **top plot** displays genetic (measured as the Euclidean distance on the two first PCs) and geographic distances over all pairs of samples (N = 5,151). In the **bottom plots**, all pairs are partitioned on their type: **(a)** both samples from the Western cluster, **(b)** each sample from a different cluster, and **(c)** both samples from the Eastern cluster.


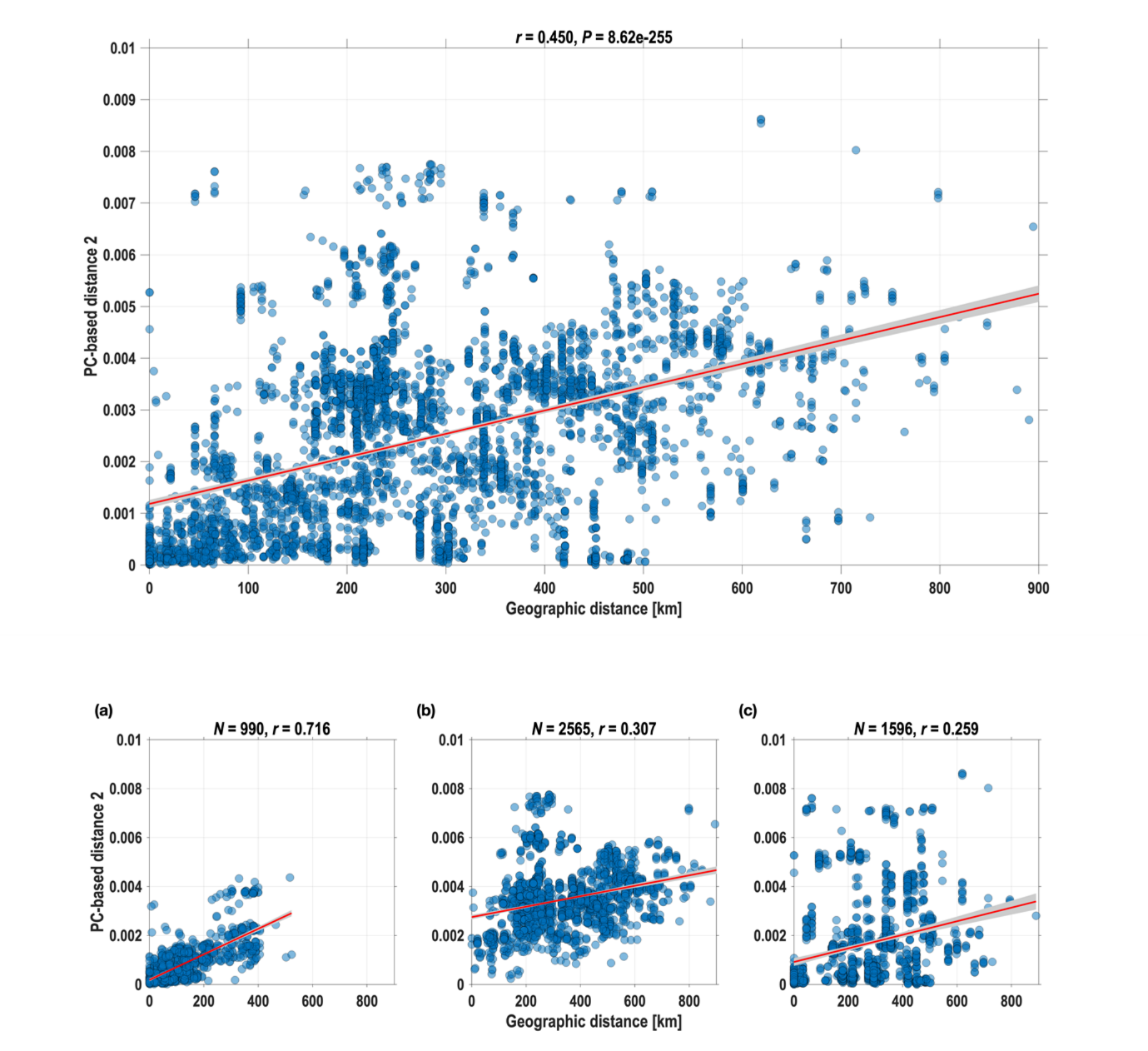


## Figure S6. PCA of the test population using each of the 4 panels of 96 selected SNPs

The 4 PCA plots here below show the distribution of the 63 test samples (20 Algerian bees + 13 M lineage + 30 C lineage) on the first 2 principal components obtained using the 96 selected SNPs with each configuration (from top to bottom, left to right: GI1, GI2, EN1 and EN2). The 4 scree plots that follow correspond to the each of 4 PCA. In all cases the top-2 PCs explain a much larger proportion of variance than the subsequent PCs.


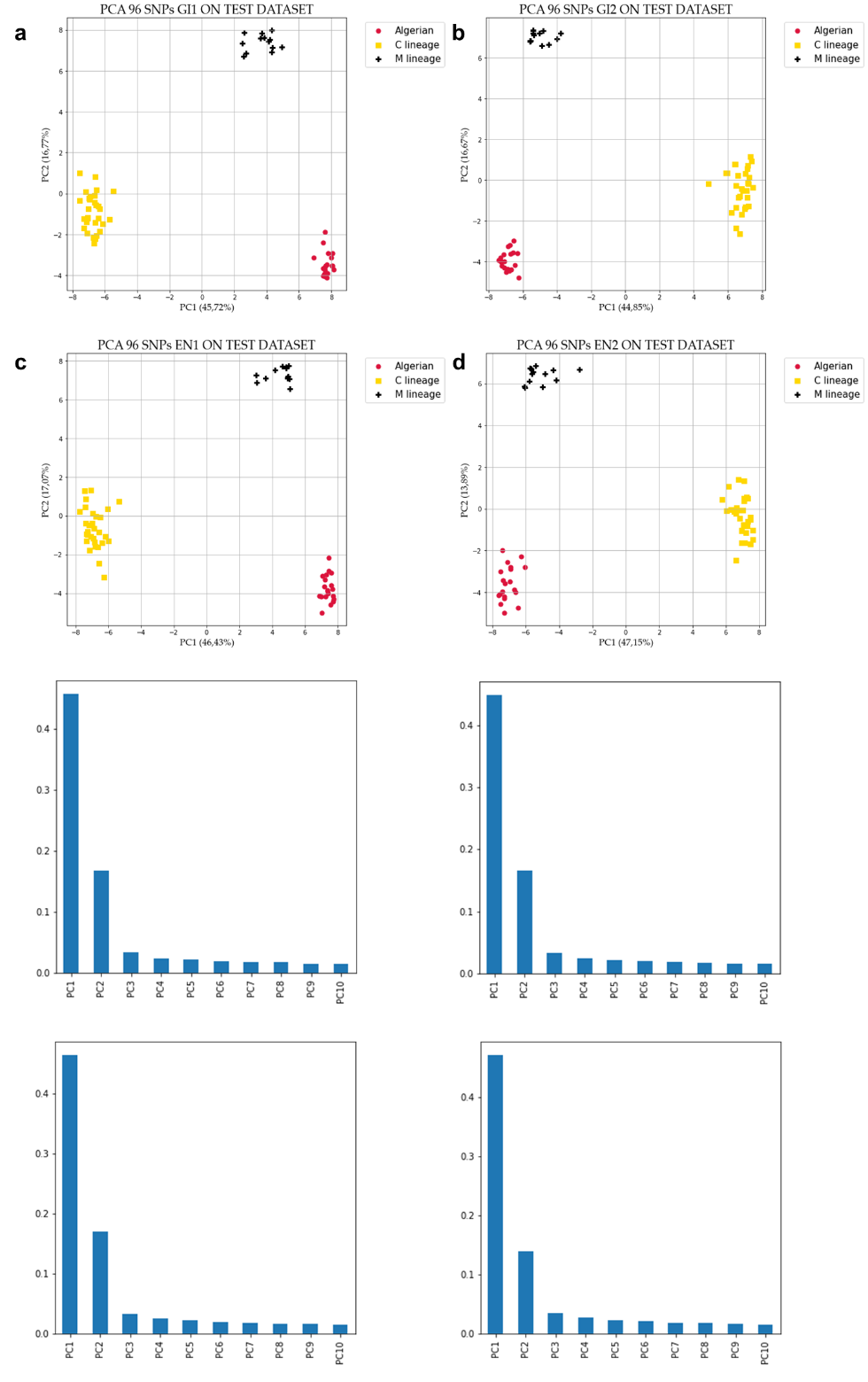


## Figure S7. Prediction performance of the list of 96 selected SNPs against 1,000 lists of randomly-chosen SNPs

We compared the selected panel of 96 SNPs (GI1) to 1,000 randomly-selected SNPs. After using each of these random panels to repeat 50 times the training of a RF classifier ({Algerian; non-Algerian}) and its application to assignment of the 63 test samples, we recorded 3 parameters: (**a**) the average classification accuracy of the test samples over the 50 runs, (**b**) the minimum probability of being labelled Algerian for Algerian bees, and (**c**) the maximum probability of being labelled Algerian for non-Algerian bees.

The values of these parameters obtained with the selected panel of 96 SNPs are show in red below each histogram.


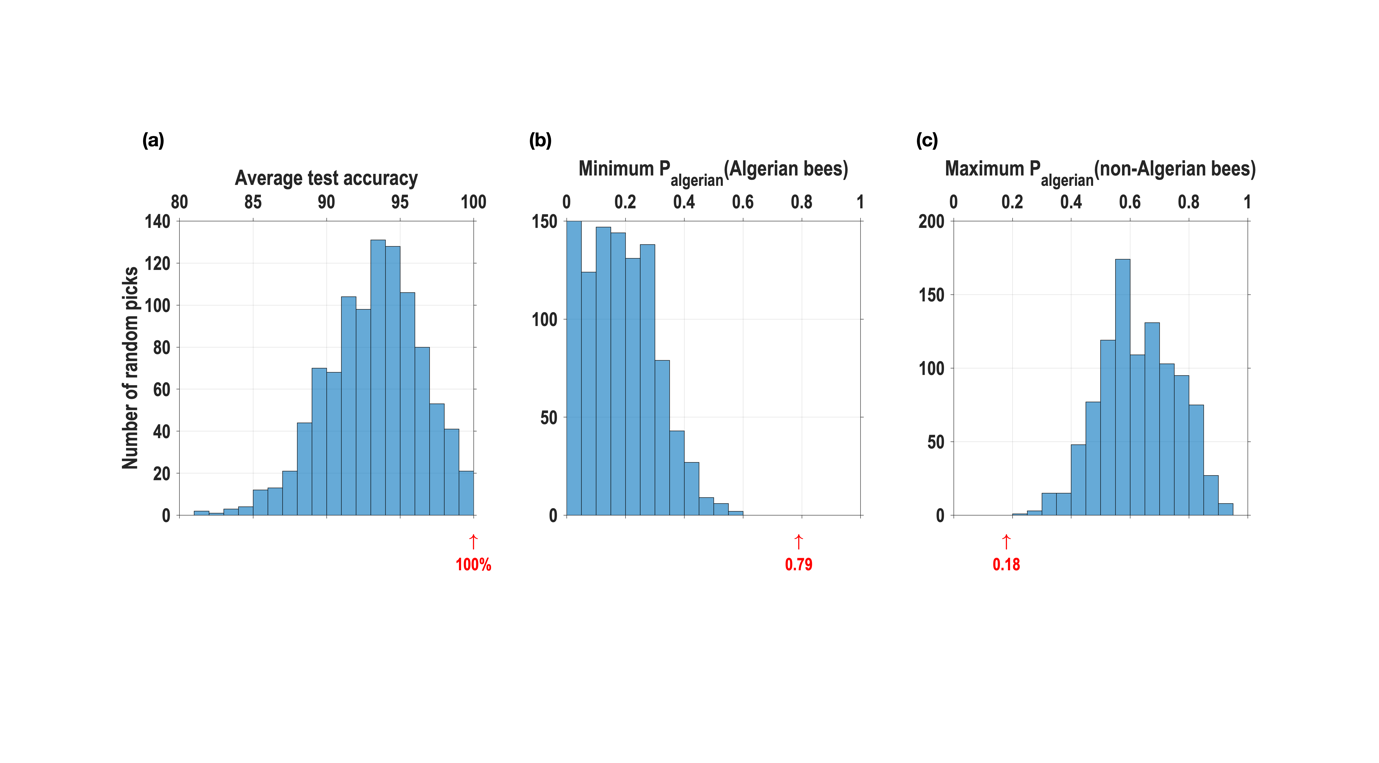


## Figure S8. Assignment probability of Algerian bees and simulated hybrids

We report here the probability of a test sample being labelled “Algerian” by a Random Forest classifier whose training set did not include simulated hybrids, for 20 Algerian bees and 320 simulated hybrids: 4 types of crosses (F1 and 3 back-crosses – BC) on 40 per type of cross.


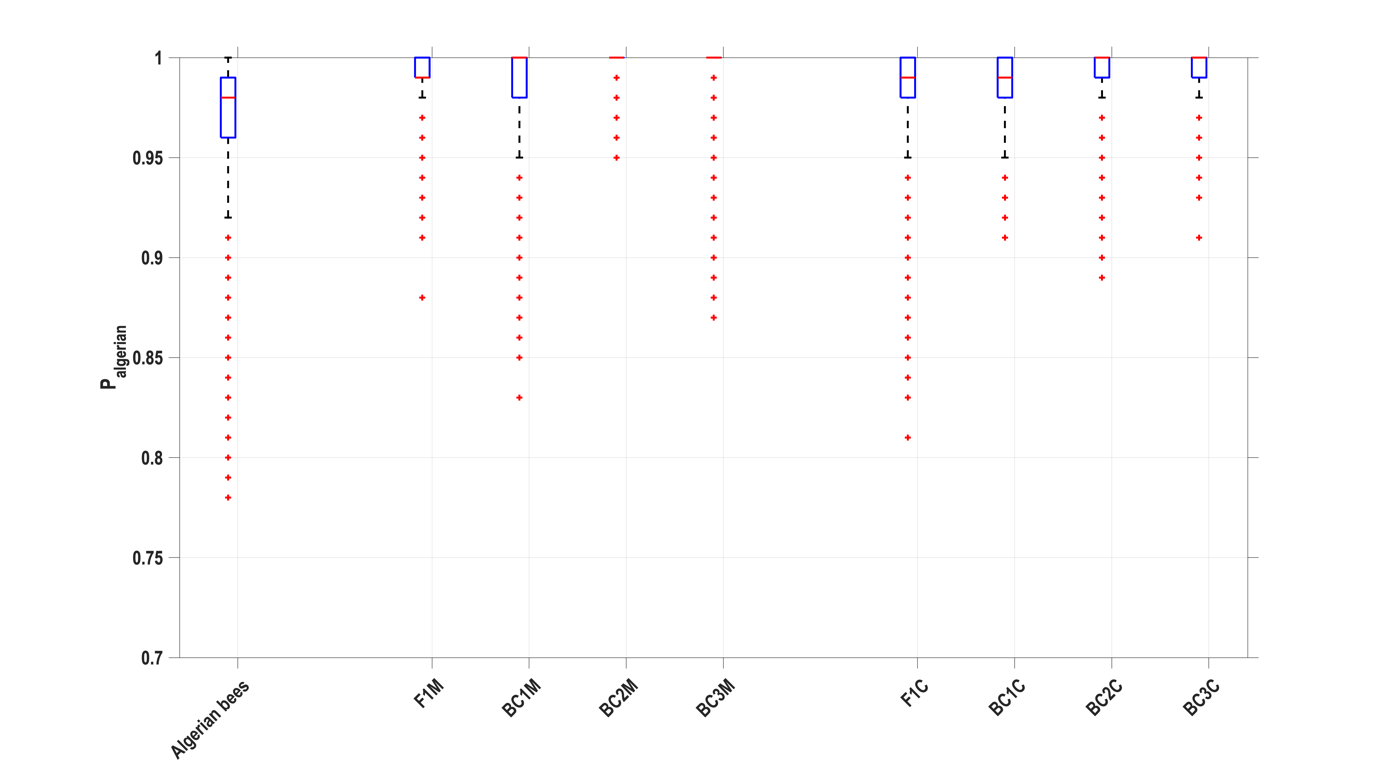


## Figure S9. Assignment probabilities, using the panel of 96 AIMs vs. using all variants

We report here the probability of a test sample being labelled “Algerian” by a Random Forest classifier trained on a population that includes simulated hybrids, using either all variants (blue boxes) or the 96 selected AIMs (yellow boxes), per population or per type of hybrid.


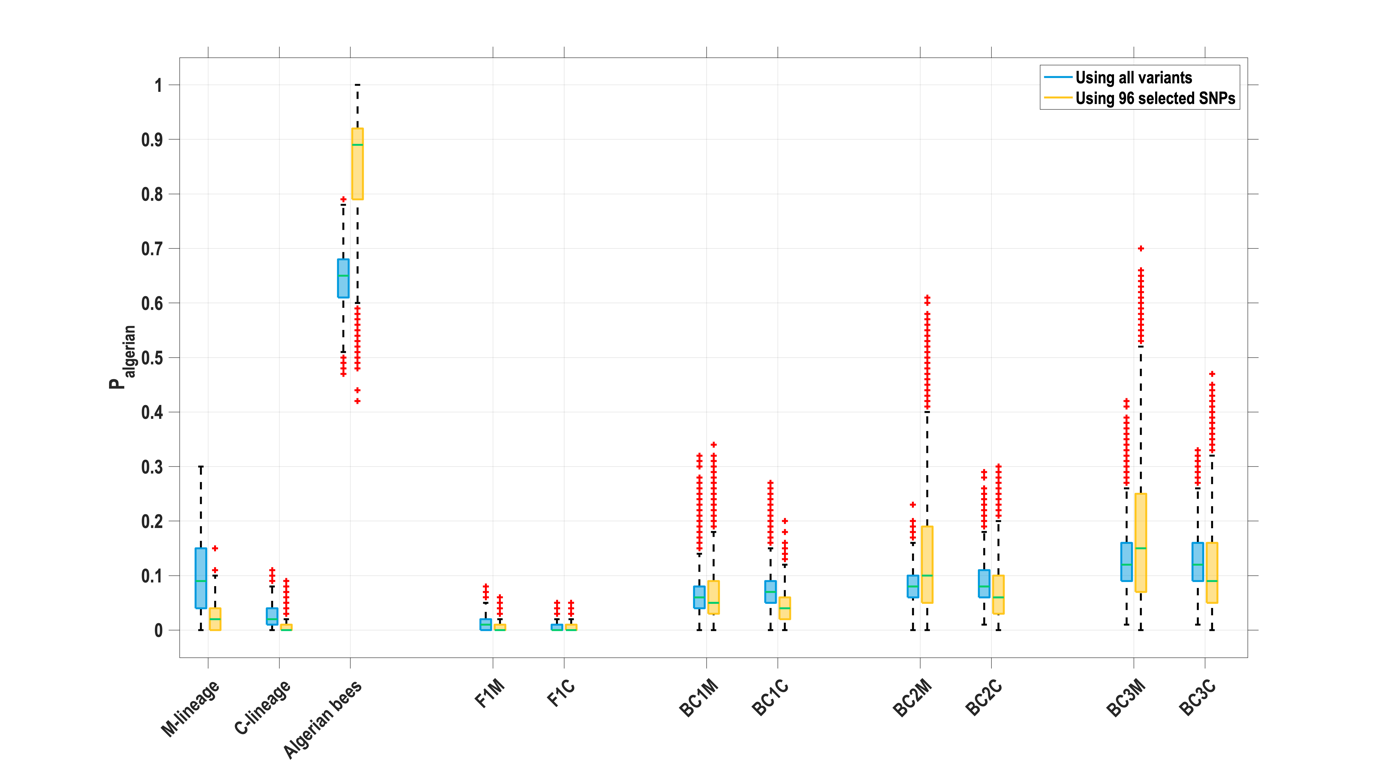

Supplement: Supplementary file 1 — Additional file 1: Figure S1. Pictures of A. m. sahariensis (a) and A. m. intermissa (b). Figure S2. Distribution of IBD kinship between pairs of Algerian samples. The distribution of the values of IBD kinship over 11,325 pairs of Algerian samples (N = 151) is shown in this figure. The Y axis gives the number of pairs in each bin on a logarithmic scale (a value of 1, 2 or 3 respectively means 1, 10 or 100 pairs in the corresponding bin). The red dashed line indicates the retained cut-off (0.3) to define close-related samples. For pairs beyond that cut-off value, we retained only one member in the final set (see Additional file 2: Table S1). Figure S3. Estimation of cross-validation (CV) error for 50 runs of ADMIXTURE for 2 ≤ K ≤ 12, considering Algerian and reference specimens. Among the K values with the lowest CV values, K = 4 stands out as having a significant mode that comprises 48 out of 50 runs. This mode also has the lowest mean CV value from the ADMIXTURE runs. The admixture plot for K = 4 is given along with plots for 2 ≤ K ≤ 6. Figure S4. Estimation of cross-validation (CV) error for 50 runs of ADMIXTURE for 2 ≤ K ≤ 12, only considering Algerian specimens. Figure S5. Scatter plots of genetic vs. geographic pairwise distances. The top plot displays genetic (measured as the Euclidean distance on the two first PC) and geographic distances over all pairs of samples (N = 5151). In the bottom plots, all pairs are partitioned on their type: (a) both samples from the Western cluster, (b) each sample from a different cluster, and (c) both samples from the Eastern cluster. Figure S6. PCA of the test population using each of the four panels of 96 selected SNPs. The four PCA plots show the distribution of the 63 test samples (20 Algerian bees + 13 M lineage + 30 C lineage) on the first two principal components obtained using the 96 selected SNPs with each configuration (from top to bottom, left to right: GI1, GI2, EN1 and EN2). The four scree plots correspond to the e [file 12711_2023_864_MOESM1_ESM.docx]
